# The economic burden of inpatient paediatric care in Kenya: household and provider costs for treatment of pneumonia, malaria and meningitis

**DOI:** 10.1186/1478-7547-7-3

**Published:** 2009-01-22

**Authors:** Philip Ayieko, Angela O Akumu, Ulla K Griffiths, Mike English

**Affiliations:** 1 Kenya Medical Research Institute/Wellcome Trust research Programme P.O. Box 43640-00100 GPO, Nairobi Kenya; 2 Health Policy Unit, London School of Hygiene and Tropical Medicine, Keppel Street, London WC1E 7HT, UK; 3 Department of Paediatrics, University of Oxford, John Radcliffe Hospital, Headington, Oxford, UK

## Abstract

**Background:**

Knowledge of treatment cost is essential in assessing cost effectiveness in healthcare. Evidence of the potential impact of implementing available interventions against childhood illnesses in developing countries challenges us to define the costs of treating these diseases. The purpose of this study is to describe the total costs associated with treatment of pneumonia, malaria and meningitis in children less than five years in seven Kenyan hospitals.

**Methods:**

Patient resource use data were obtained from largely prospective evaluation of medical records and household expenditure during illness was collected from interviews with caretakers. The estimates for costs per bed day were based on published data. A sensitivity analysis was conducted using WHO-CHOICE values for costs per bed day.

**Results:**

Treatment costs for 572 children (pneumonia = 205, malaria = 211, meningitis = 102 and mixed diagnoses = 54) and household expenditure for 390 households were analysed. From the provider perspective the mean cost per admission at the national hospital was US $95.58 for malaria, US $177.14 for pneumonia and US $284.64 for meningitis. In the public regional or district hospitals the mean cost per child treated ranged from US $47.19 to US $81.84 for malaria and US $54.06 to US $99.26 for pneumonia. The corresponding treatment costs in the mission hospitals were between US $43.23 to US $88.18 for malaria and US $ 43.36 to US $142.22 for pneumonia. Meningitis was treated for US $ 189.41 at the regional hospital and US $ 201.59 at one mission hospital. The total treatment cost estimates were sensitive to changes in the source of bed day costs. The median treatment related household payments within quintiles defined by total household expenditure differed by type of facility visited. Public hospitals recovered up to 40% of provider costs through user charges while mission facilities recovered 44% to 100% of costs.

**Conclusion:**

Treatments cost for inpatient malaria, pneumonia and meningitis vary by facility type, with mission and tertiary referral facilities being more expensive compared to primary referral. Households of sick children contribute significantly towards provider cost through payment of user fees. These findings could be used in cost effectiveness analysis of health interventions.

## Background

The 4^th ^Millennium Development Goal (MDG4) is to reduce child mortality by two-thirds between 1990 and 2015 [1]. Kenya is currently not on track to reach this goal [2]. In 1990 the Kenyan under-five mortality rate was reported as 97 deaths per 1000 live births, but in 2006 it had increased to 121 deaths per 1000 live births [3]. Kenya is thus among the ten countries with least progress towards MDG 4 and substantial changes are needed to reach the goal of 32 deaths per 1000 live births in 2015 [3].

The most common causes of deaths in Kenyan children after the neonatal period are pneumonia, diarrhoea, measles, malaria, and malnutrition or a combination of these conditions [4]. It has been estimated that 63% of global childhood deaths could be prevented if interventions of proven efficacy were universally available [5]. These interventions are a mixture of prevention strategies, such as vaccination and insecticide-treated bed nets, and case management. Case management is currently undertaken at different levels of the Kenyan health system, from outpatient clinics to tertiary hospitals. The increase in under-five mortality during the past decade can be plausibly linked to limited access to case management, sub-optimal quality of care at the facilities and late health care seeking behaviour [6-8].

A possible barrier to accessing case management care in Kenya is the costs of treatment, but knowledge of such costs is limited. The objective of this study is therefore to describe the costs associated with treatment of pneumonia, malaria and meningitis among children admitted to Kenyan hospitals. The viewpoint of the analysis is the public health sector as well as households. Although care for children under five years is officially free of charge in Kenya, households frequently pay for hospital stay and/or drugs and supplies (henceforth all termed user fees). These payments made by poor households directly to service providers are high and continue to grow [9]. Hence, an objective of the study was to estimate the proportion of total treatment costs covered by households.

Analysis of meningitis and pneumonia treatment costs was undertaken because a proportion of these cases can be prevented by *Haemophilus Influenzae *type b (Hib) and pneumococcal vaccines. Recent projections show that introduction of Hib and pneumococcal conjugate vaccines could reduce the global burden of pneumonia severe enough to require hospitalization by half while impacting significantly on all cause childhood mortality [10,11]. Treatment cost data of the present study can be integrated into cost-effectiveness analyses of these vaccines. Malaria was included to achieve a more comprehensive overview of the costs of childhood illnesses and to provide a comparator to pneumonia, as these two diseases are the primary cause of inpatient admissions in Kenyan children [4].

## Methods

### Study sites and sample selection

Resource utilisation data were collected from a sample of 7 hospitals selected purposefully from the facility list of the Ministry of Health. For the public health sector, the selection strategy aimed to ensure representation of large tertiary hospitals (1 of 2 possible sites), medium sized regional hospitals (1 of 7 possible sites), and smaller district hospitals (3 of 65 possible sites), with representation of different geographic and climatic regions within Kenya. In addition, we selected 2 not-for-profit (mission) hospitals providing first referral level services equivalent to district hospitals. The selected sites are summarized in Table 1.

**Table 1:** Location and characteristics of the 7 study hospitals

Hospital identifier (location)	Hospital type (referral level)	Number of beds (bed occupancy)	Malaria endemicity	Funding source	User charging policy
H1 (Nairobi province)	National (tertiary)	1520 (138%)	Low	Government budgetary allocation, user fees and occasional donations.	To offer free health care services to all children under 5 years.
		
H2 (Rift valley province)	Provincial (secondary)	453 (80%)	Moderate		
		
H3 (Nyanza province)	District (primary)	234 (80%)	High		
		
H4 (Central province)	District (primary)	208 (120%)	Moderate		
		
H5 (Eastern province)	District (primary)	182 (108%)	Moderate		

H6 (Rift valley province)	Mission (primary)	308 (49%)	Moderate	User fees (approx 80% of the budget), government seconded staff, donations (unpredictable and occasional).	Operate waiver and credit facilities for patients not able to pay.
		
H7 (Rift valley province)	Mission (primary)	160 (59%)	Moderate		

The study population included all admissions under 5 years of age with a clinical diagnosis of pneumonia, malaria or meningitis. Based on the mean costs of paediatric admissions reported in two previous studies [12,13] we estimated that 30 patients for each diagnostic group would allow reporting of results around a mean cost of US$ 100 with estimated precision represented by a standard error of US$ 6 and 95% confidence interval of ± US$ 12. Hence, we aimed to collect data on resource use from a sample consisting of at least 30 patients with a diagnosis of malaria and 30 patients with pneumonia per site. For meningitis, known to be a considerably less common diagnosis, the aim was to obtain data from at least 30 cases across all sites.

### Data collection

Data were collected from November 2004 to October 2005 using two methods: review of medical records and interviews with caretakers. The aim was to recruit children prospectively over a 6 week period at each site with the option to include cases identified retrospectively from those admitted in the immediate preceding months if case numbers were small, particularly likely for meningitis. To ensure accuracy and uniformity across sites an investigator (AA) visited each hospital for the first 10 days of data collection and trained a nurse in the use of two data collection tools. At the end of the study period AA returned to the sites and checked the data quality. We selected nurses for data collection with careful attention to the task of abstracting information from medical records. To minimise the potential for reporting bias as a result of interaction between health worker and caregiver, study nurses did not perform regular clinical work during the data collection period. In most cases we recruited nurses on annual leave.

With the first tool the nurse collected patient specific resource utilisation data as described in patient records. The process involved recording the length of hospital stay (by type of department), the quantity of pharmaceuticals and supplies used by each patient, and the use of diagnostic tests and other specialized services. Using the second tool, the nurse completed a structured interview with the caretaker of each child. The interview was initiated during admission with the nurse asking about out-of-pocket spending on health care prior to admission, transport and costs related to the admission episode, including user fees. The facility based nurse continued collecting data on expenses incurred by caretakers on a daily basis until discharge. These included transportation of household members visiting the child and all additional out-of-pocket payments.

It was concluded from a number of pilot interviews that questions about household income generated unrealistic answers, as most caretakers were not head of households and therefore lacked knowledge about this. Instead, information on monthly household spending was collected by asking caretakers for estimates of amounts spent on food, rent, education and healthcare. To reduce recall bias caretakers were required to report on the most recent expenditure with the option of breaking down the recall period into daily, weekly or fortnightly expenditure on specified cost items. Before a cost item was recorded as unknown, caretakers were asked to enquire about expenditure on that specific item from household heads. The questionnaire used is included in additional file 1.

Caretakers who participated provided informed written consent. The Kenya National Ethical Review Committee and the WHO Ethical Committee approved the study.

### Unit costs

Unit costs were estimated in 2005 US$. The average 2005 exchange rate of US$ 0.01329 to the Kenyan shilling was used .

#### Medication costs

While the Kenya Medical Supply Agency (KEMSA) is the leading supplier of essential drugs to government health facilities, mission facilities procure their drugs from the not-for-profit Mission for Essential Drugs Supply (MEDS) and from private-for-profit distributors [14]. We mainly estimated drug unit costs from the KEMSA price list, but for drugs not included in this list and for mission facilities we used the hospitals' own purchase price lists or the MEDS list. We applied either a dose-specific cost or the full cost, depending on whether a drug was reusable or had to be discarded once it was opened and partially used. For blood transfusion the cost attributed to each episode was based on the reported costs of providing one unit of blood from the National Blood Transfusion Service (Table 2).

**Table 2:** Unit cost estimates for selected items used in the cost analysis (2005 US$)

**Investigation/Procedure**	**Unit cost** **(2005 US$)**	**Source**
Inter costal drainage	21.91	Kilifi District hospital

Blood transfusion	34.60	National blood transfusion centre

Resuscitation	3.78	Tenwek Mission Hospital

Chest X-ray	1.94	Kilifi costing, KNH

Blood count	8.26	Kilifi District hospital

Lumbar puncture laboratory supplies	1.40	NetSPEAR*

CSF Kenyatta procedures	37.80	Kenyatta National Hospital

CSF Kilifi procedures	14.69	Kilifi District hospital

Blood culture	18.36	Kilifi District hospital

HIV test	3.68	Kilifi District hospital

Ultrasound	6.05	Kenyatta National Hospital

Electrolytes	4.04	Kilifi District hospital

Glucose	3.68	Kilifi District hospital

Blood slide for malaria parasites	3.50	Kenyatta National Hospital

Other microbiology	14.69	Kilifi District hospital

Day in Kenyatta National Hospital	17.46	Guinness *et al *(2002)[16]
	
	8.85	WHO CHOICE 2005

Day in provincial hospital	13.52	Average Nganda *et al *(2003)[17] & Guinness *et al *2002[16]
	
	6.48	WHO CHOICE 2005

Day in primary referral hospital	9.57	Nganda *et al *(2003)[17]
	
	4.97	WHO CHOICE 2005

*NetSPEAR – The Network for Surveillance of Pneumococcal Disease in the East African Region

CSF: Cerebrospinal fluid

#### Diagnostic tests

There was limited information across study sites on the cost of conducting basic clinical and laboratory diagnostic investigations. We therefore used the user charge price list from Kenyatta National Hospital as well as cost estimates for conducting these investigations at the Kenya Medical Research Institute/Wellcome Trust clinical laboratories in Kilifi District Hospital. The unit cost for laboratory investigations on cerebrospinal fluid (CSF) was estimated from both sources. The (lower) estimate from Kilifi that covered only essential CSF examination was applied in most cases unless specific, additional investigations were ordered in hospitals that had the capacity to perform these tests (Table 2).

#### Costs per hospital bed day

Bed day costs represent the "hotel" component of hospital costs, i.e. excluding drugs and diagnostic tests which vary by patient, but including other costs such as personnel, buildings, food, laundry and capital costs. As illustrated by Adam *et al*. [15] one of the most important determinants of bed day costs is the hospital bed occupancy rate; the higher the occupancy rate, the lower the costs per bed day. Occupancy rates of the studied facilities are included in Table 1. It is seen that the occupancy rates at mission hospitals are considerably less than at Government facilities.

It was not possible to conduct a full micro costing at the hospitals, so we used estimates based on average bed day costs derived from two costing studies conducted around the time of our data collection. One of these studies was conducted at Kenyatta Hospital, which was also a hospital included in our sample [16]. For the tertiary hospital we used the estimates by Guinness *et al *(2002) and for the primary referral hospitals the values presented by Nganda *et al *(2003) [17] were used. These studies estimated provider costs and presented costs for each component of treatment, including a day in hospital. The cost per bed day in a provincial hospital was estimated as an average between these two unit costs. Adjustments were made for inflation to reflect 2005 values. For comparison, the 2005 estimates produced by the WHO-CHOICE project for different facility levels in Kenya  are presented along with the unit costs in Table 2 and these values are used in a sensitivity analysis. It is seen that the WHO-CHOICE estimates are approximately half as much as found in the case studies.

#### Caretaker time

All hospitalised children were accompanied by an adult caretaker. The total time lost by caretakers was estimated by adding the time spent seeking health care prior to admission and the duration of inpatient stay. Two main methods that are generally accepted for attaching a monetary value to time lost due to morbidity and health care seeking. With the human capital approach a focus is placed on the impact of lost work time and the gross wage is used to place a value on time [18]. In the friction cost approach the gross wage is still used to value time, but unemployment is taken into account with the argument that output may be made up on return to work or by replacing workers from the unemployed [18]. Hence, the productivity costs estimates are lower in the friction than the human capital approach. However, since the gross wage is not a meaningful term in a subsistence economy like rural Kenya, none of these approaches are directly applicable. We used instead an estimate by Larson *et al *in a study on the cost of uncomplicated childhood fevers to Kenya households [19]. Based on a review of existing literature on poverty, adult daily income and wages in Kenya, Larson et al. concluded that US$ 1.00 per day provides a reasonable estimate of the average monetary value of caretaker time. This value is less than the 2001 average daily wage of US$ 1.31 of female horticultural workers reported by Dolan and Sutherland [20]; thus adjusting for unemployment and the fact that a relatively large percentage of women work in subsistence agriculture [18]. To assess the importance of this value we reduced it by half in the sensitivity analysis.

### Estimation of total costs

Individual patient resource use data were coupled with unit cost estimates to generate a patient specific cost estimate for meningitis, pneumonia and malaria cases. To obtain the average total cost of treatment per case we added up the cost of drugs, diagnostic investigations and hospital stay costs. Average treatment costs at the national hospital and pooled data from the three district hospitals were compared using t-test. The 95% confidence interval for difference in arithmetic mean between treatment groups is likely to be very similar whether t-test based methods or bootstrapping is used, even with moderate sample sizes and highly skewed cost data [21].

For the subset of children with caretaker interview information we calculated direct caretaker expenses by adding up pre-admission treatment, transport costs, user fees, and out-of-pocket costs for items not supplied by the hospital. When calculating the sum of provider and household costs we excluded user charges to avoid double-counting.

## Results

### Sample characteristics

In total, we reviewed 572 records (418 prospective, 154 retrospective) of children with pneumonia, malaria and meningitis. 90% of the children had only a single diagnosis: 211 had malaria, 205 had pneumonia, and 102 had meningitis. The remaining 54 children had more than one of the diagnoses, with 41 of these having a combined diagnosis of malaria and pneumonia. We excluded the 13 children with other co-morbidity diagnoses from further analyses.

Preliminary analysis showed that data collected prospectively by the study nurse during the 6 week period were not different to those of retrospective cases and we therefore pooled and analysed the data together. We obtained interviews with 393 (94%) out of the 418 caretakers for whom we had prospective treatment cost data. Age and sex distributions for the different diagnoses were similar. The median age (interquartile range, IQR) of children in the entire sample was 12 months (5.5–24) and 323 (56%) children were boys.

Meningitis diagnoses were over represented within three hospitals; 43 out of the 102 cases were treated at the national hospital and a further 46 at either the regional hospital or a single district hospital. Table 3 shows that the number of patients with a diagnosis of malaria or pneumonia ranged from 17 to 49 cases per site. Overall, 48 (8.4%) children died while admitted at the facilities. The disease specific case fatality rates were 5% for malaria, 10% for meningitis and 11% for pneumonia.

**Table 3:** Mean cost for malaria, pneumonia and meningitis treatment in 2005 US$ among children admitted to seven Kenyan hospitals

Hospital	Diagnosis	No of patients	Average length of stay (days)	Mean drug costs	Mean cost of investigations	Mean bed-day cost	Mean (SD) health sector cost per patient*	Median (IQR) health sector cost*
*National hospital*								

H1	Malaria	27	4.3	4.07	16.49	75.03	95.58 (78.27)	78.72 (40.36–125.41)

	Pneumonia	49	8.2	15.17	18.70	143.26	177.14 (206.76)	117.40 (58.97–201.90)

	Meningitis	43	12.8	24.33	36.95	223.37	284.64 (239.38)	222.60 (159.68–317.06)

*Provincial hospital*								

H2	Malaria	29	4.7	8.68	9.77	63.40	81.84 (73.60)	58.73 (37.46–103.60)

	Pneumonia	31	6.6	6.57	3.53	89.67	99.26 (71.14)	71.94 (55.26–109.75)

	Meningitis	24	11.7	18.41	13.29	157.71	189.41 (141.58)	165.31(126.24–237.23)

*District hospitals*								

H3	Malaria	44	4.8	3.06	26.25	45.91	75.22 (42.09)	75.13 (36.33–102.84)

	Pneumonia	29	4.8	2.23	8.44	45.89	56.55 (60.37)	41.42 (23.75–69.89)

H4	Malaria	25	4.7	4.11	5.85	45.19	55.16 (30.67)	46.64 (34.13–67.76)

	Pneumonia	30	6.7	2.88	4.94	64.14	71.96 (29.38)	56.00 (52.65–84.09)

H5	Malaria	25	3.8	2.68	8.51	36.00	47.19 (43.16)	37.98 (33.21–45.01)

	Pneumonia	17	4.2	4.29	9.79	39.98	54.06 (33.82)	50.65 (30.90–66.61)

	Malaria and pneumonia	20	4.5	2.42	8.03	43.08	53.53(34.41)	79.00(34.91–121.16)

*Mission hospitals*								

H6	Malaria	30	4.5	13.15	31.62	43.40	88.18 (59.92)	72.82 (43.65–113.83)

	Pneumonia	30	7.8	32.15	35.39	74.68	142.22 (103.39)	140.49 (51.04–185.23)

	Meningitis	22	11.8	44.03	44.85	112.71	201.59(126.41)	162.00(115.16–248.14)

H7	Malaria	31	3.1	6.34	6.55	29.34	42.23 (23.60)	36.46 (32.80–46.45)

	Pneumonia	19	3.4	3.05	7.56	32.75	43.36 (29.75)	35.53 (31.96–51.01)

*Excluding pre-admission costs, caretaker time and transport costs, but including revenues generated from user fees.

### Resource use

Children in the study were admitted to hospital after a median duration (IQR) of 3 days (2–6 days) following the onset of symptoms. Approximately half of the caretakers sought care from other sources before going to hospital. The length of stay in hospital ranged from 1 day to 68 days with a median (IQR) of 5 days (3–8 days). The admission prescriptions across all sites frequently included non essential drugs which were not related to the admission diagnosis. All children with pneumonia as a single diagnosis received at least 4 drugs. Over half the children with malaria and 78% of the meningitis cases received 5 or more drugs. For investigations, at least one basic diagnostic test was ordered in 91% of the children during inpatient stay. The most common request was a blood slide for malaria parasites. 27% of the malaria cases and 21% of the pneumonia cases had more than 2 tests done. Blood culture was available at only two sites and HIV testing was rarely done although it was reportedly available across sites.

### Provider cost estimates

**Figure 1:**
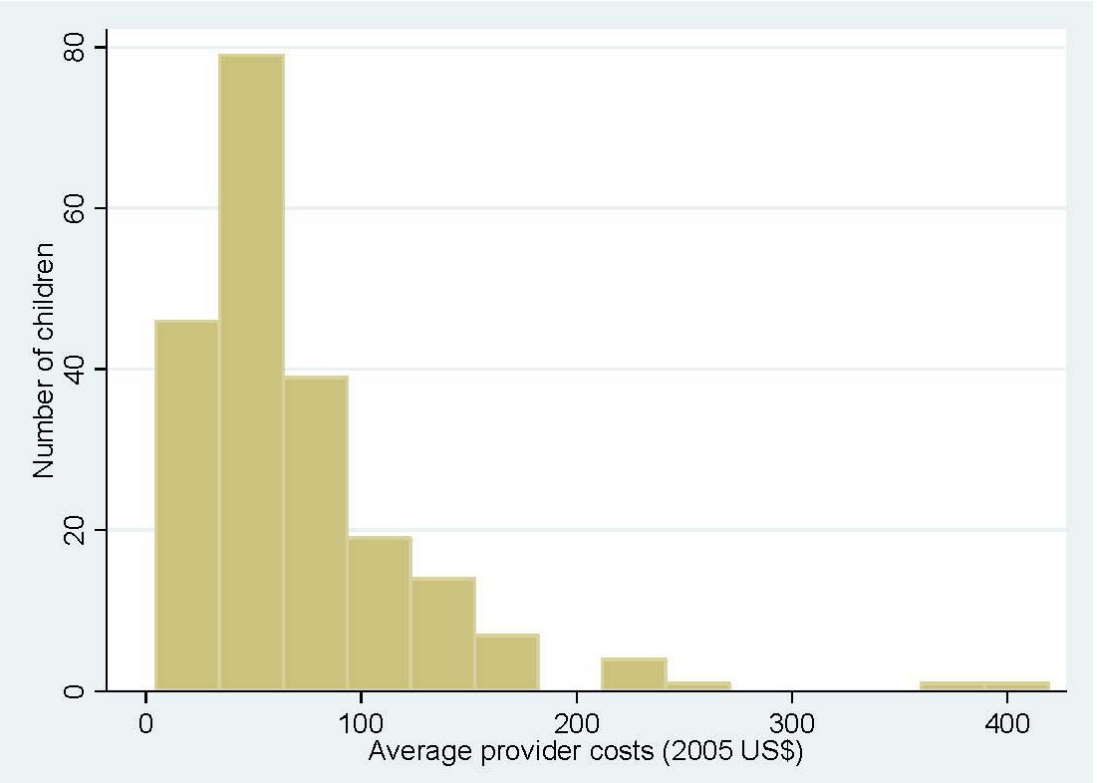
**Distribution of provider costs for treating malaria (Pooled data from all facilities)**.

Figure 1 shows the right-skewed distribution of provider costs for children with malaria. A similar pattern was seen for pneumonia and meningitis costs. We therefore report the median cost and inter-quartile range in Table 3 as most appropriately representing the central tendency and range of costs, but also the mean costs as these are the most useful for estimating total costs for any number of patients [21]. At the seven facilities the mean total provider costs for treating a case of malaria ranged from US$ 42.23 to US$ 95.58, while the cost of an episode of pneumonia was between US$ 43.36 and US$ 177.14. At the national hospital the mean treatment costs of meningitis were US$ 290.42. At the other two hospitals where children with meningitis were seen the mean treatment costs were US$ 189.41 and US$ 205.74 per case.

The mean provider costs of investigations and drugs for pneumonia patients were consistently higher at the tertiary referral hospital compared to the 3 public district hospitals (Table 4). On the whole, there was a difference in total costs for children with pneumonia (P < 0.0001) and malaria (P = 0.004) at the national hospital and the district facilities. Similarly, the costs of treatment at one of the mission hospitals with more advanced diagnostic facilities approximated that at the tertiary referral level and differed significantly from other district hospitals (P < 0.0001).

**Table 4:** Mean cost* for pneumonia and malaria treatment at national referral hospital and pooled data from the 3 district hospitals (2005 US$)

Cost category	Facility type		Difference between facilities (95%CI)	P value
			
	National hospital	District hospitals		
*Pneumonia*				

Investigations	18.70	7.36	11.34 (5.10–17.59)	< 0.001

Drug cost	15.17	2.95	12.23 (6.46 – 17.99)	< 0.001

Hospital stay	143.27	51.77	91.50 (48.21–134.78)	< 0.001

Total	177.14	62.08	115.06 (66.54–163.59)	< 0.001

*Malaria*				

Investigations	16.49	16.10	0.38 (-8.15–8.91)	0.93

Drug costs	4.07	3.24	0.83 (-1.0–2.66)	0.37

Hospital stay	75.03	43.08	31.95 (13.56–50.33)	<0.001

Total	95.58	62.42	33.15 (10.84 – 55.47)	0.004

*Excluding pre-admission costs, caretaker time and transport costs

### Caretaker costs

Caretakers spent an average of 1 hour and 49 min (range, 15 min to 10 hours) travelling to seek health care services for their sick children. Consequently, an average round trip would last 3 hours and 38 min, or approximately 0.5 working days. Table 3 presents the average length of stay (days) according to facility and diagnosis. The average time caretakers spent in caring for an admitted child regardless of diagnosis was 6.5 days (SD = 7.5).

All the caretakers, both in government and mission hospitals reported that they were required to pay a user charge. For those who used public facilities 89% reported making either partial or complete payments of the total amount required. The remaining cases were waived by the hospitals with most of the waivers being at the national hospital. At one mission hospital all the caretakers reported having made payments while the second hospital waived 2 cases and discharged 4 children whose caretakers were to pay for the services later.

At least 25% of the children admitted at the tertiary hospital were still in the ward waiting for relatives to be able to pay user fee bills on average 4 days after being medically discharged at a cost of US$17.46 to the provider and a charge of US $ 5.32 to the household per each extra day spent in hospital. The longest stay by a patient awaiting administrative discharge at this institution was 22 days. At the district hospitals it was found that 10% of admissions remained in the wards 2 to 3 days after medical discharge because the family did not have funds to pay the bill. The mission hospitals on the other hand operated an early discharge system, at times offering credit terms of payment to households with difficulties in raising the required funds.

Payment of user fees by caretakers resulted in recovery of a substantial proportion of the treatment costs. Approximately 44% to 100% of provider costs at the mission hospitals were recovered from user fees. At the government tertiary referral facility an average of US$ 65.10 was recovered per admission accounting for approximately 40% of total costs. At public district hospitals the costs recovered from households amounted to between US$ 6.1 and US$ 19.66 per child admitted, which is approximately 15% of the treatment costs.

**Figure 2:**
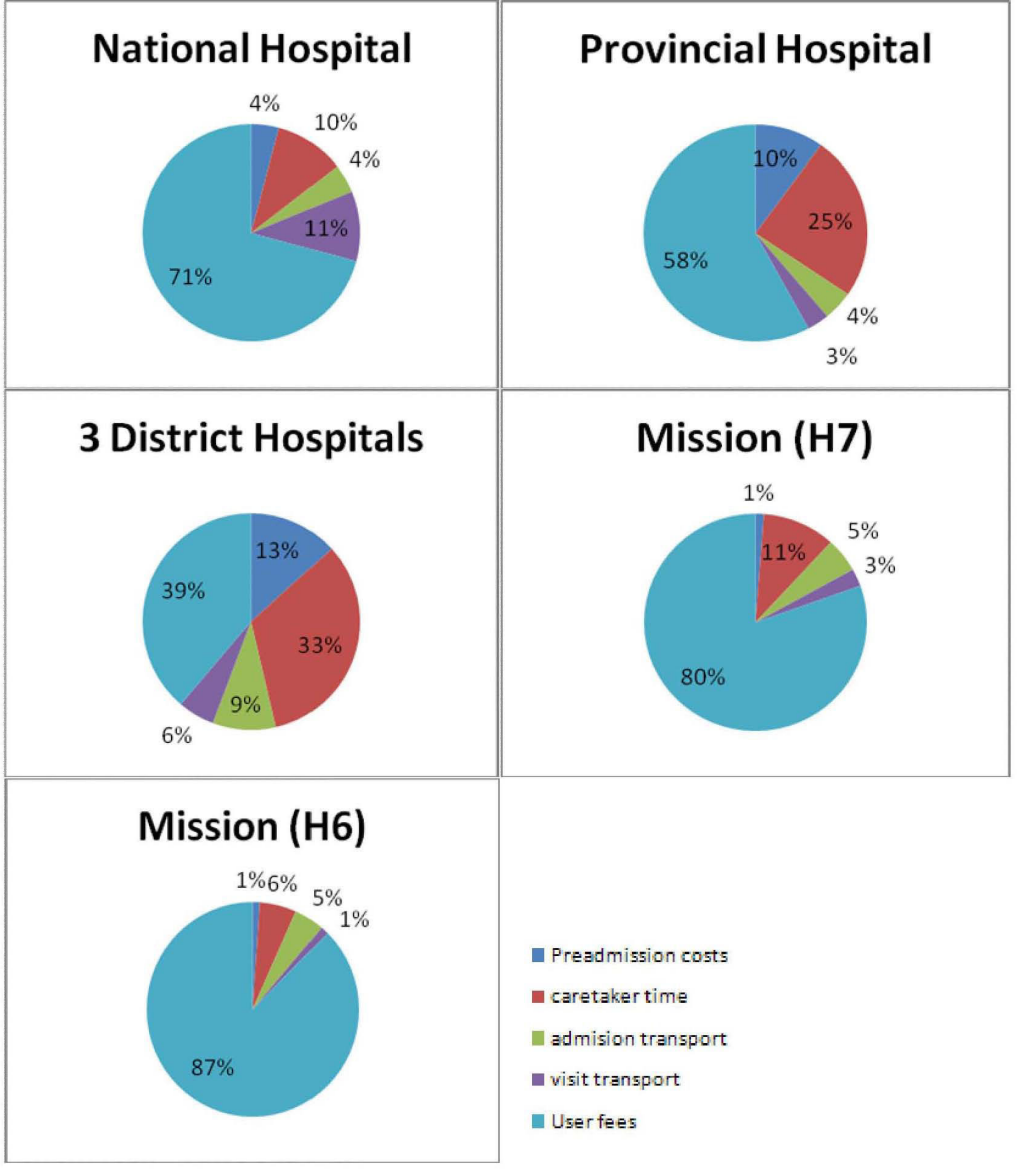
**Distribution of caretaker cost according to expense category and facility type**.

The distribution of household costs according to expense category and facility type is illustrated in Figure 2. The relative contribution of user charges to total household spending on healthcare is lower in public district facilities compared to mission hospitals and the tertiary referral hospital. Transportation costs associated with completing referrals contribute significantly to household costs at tertiary referral level (11% of total costs). The reported sources of funds for payment of inpatient care was personal savings (64%), borrowing to repay later (8%), applications for waivers (5%), and donation from friends and relatives (4%).

Table 5 compares the median household out of pocket costs with the median reported monthly household expenditure within household expenditure quintiles (lower, middle and upper tertiles) at different facilities. The households in the lowest expense category within mission facilities paid a median user charge of US$ 61.81 compared to their median total household expenditure of US$ 44.11 per month. The median user charge paid across expenditure quintiles were comparable within facility types, apart from the national hospital where households in the lowest expenditure category made lower payments (US$28.94) compared to those in the middle (US$ 41.33) and upper (US$ 52.63) expenditure tertiles. The lowest median payment for transportation was within the lowest expenditure tertile (US$ 1.46) at secondary referral facility while the highest median payment was (US$ 10.10) within the highest expense group at the national referral. Preadmission treatment costs were generally low (median payments from US $ 0.03 to 4.39) and caretaker time costs were comparable across facilities and expenditure groups (median time costs US $5.29 to 9.00).

**Table 5:** Median monthly household expenditure and median user charge payments by household expenditure tertiles within different facility types

	*National referral hospital*	*Secondary (provincial) referral*	*District hospitals*	*Missionary hospitals*
**Lower expenditure tertile**				

Number of households, n	28	16	58	26

Median household monthly expenditure (US$)	55.93	46.74	35.33	44.11

Median user charge payment (US$)	28.94	15.48	5.45	61.81

Median transport costs (US$)	6.38	2.99	1.86	3.99

Median preadmission cost (US$)	0.53	1.53	1.26	0.03

Median time costs (US$)	6.91	6.5	6.5	6.58

				

**Middle expenditure tertile**				

Number of households, n	27	16	58	27

Median household monthly expenditure (US$)	96.80	73.76	69.12	96.35

Median user charge payment (US$)	41.33	17.88	6.25	53.16

Median transport costs (US$)	13.29	1.46	1.86	3.32

Median preadmission cost (US$)	2.69	2.75	0.70	0.93

Median time costs (US$)	7.00	8.25	5.29	6.00

				

**Upper expenditure tertile**				

Number of households, n	27	16	58	24

Median household monthly expenditure (US$)	169.67	122.02	140.62	191.27

Median user charge payment (US$)	52.63	17.01	7.97	60.00

Median transport costs (US$)	10.10	1.59	2.26	6.51

Median preadmission cost (US$)	4.39	4.45	1.40	1.13

Median time costs (US$)	6.50	5.63	6.0	9.00

### Total costs

The societal cost (direct costs plus caretaker time costs) for meningitis was not calculated because 70% of the meningitis data was retrospective and lacked caretaker information. Using prospective data only the mean total cost for malaria treatment was US$ 135.57 and US$ 197.54 for pneumonia at the national hospital. Hence, within this facility pre-admission costs, transport costs and the opportunity costs of caretaker time amounted to US$ 16.12 for malaria and US$ 27.28 for pneumonia, representing 12% and 14% of total costs, respectively. At public district hospitals the mean total costs were US$ 75.21 for malaria and US$ 74.64 for pneumonia, and at the mission hospitals total costs were US$ 89.59 and US$ 135.26 for malaria and pneumonia, respectively. The costs of pre-admission, transport and time were on average US$ 12.49 and US$ 11.93 for malaria and US$ 12.54 and US$ 18.82 for pneumonia in public district hospitals and mission hospitals, respectively.

### Sensitivity analysis

The base case results were relatively insensitive to one-way variation in our major assumption on the value of caretaker time. Assuming that we had overestimated the value of caretaker time in the base case analysis by 100%, the corresponding effect would be a 2% reduction in costs at the national referral hospital and a 5% reduction at district facilities. However, the treatment cost estimates were sensitive to the source of bed day costs (published costing case studies or WHO CHOICE values) used. The costs of treatment for all the 3 diagnoses were lower across facilities when bed day cost values used in base case analysis were replaced by WHO CHOICE values. Across the seven hospitals the treatment costs were between 23% and 40% lower for malaria and 25% to 47% lower for pneumonia when WHO CHOICE estimates were used.

## Discussion

Our findings indicate that there exist significant differences in the provider costs of treating pneumonia, malaria and meningitis in childhood within public and mission facilities in Kenya. There are further cost differences within the public sector depending on the level of the facility and presenting diagnosis. Households subsidise provider costs partially through payment of user charges in the public sector and within the mission sector the entire provider costs may be passed on to households.

The difference in costs of treating malaria, pneumonia and meningitis according to type of facility depends to a certain extent on existing diagnostic capacity. For malaria, a disease with a standard and widely available diagnostic approach, the cost of investigation and drug treatment in the national hospital was not significantly different from the district hospitals. However, for pneumonia the costs were higher at the national hospital and one mission facility. In this mission facility with advanced diagnostic capacity the cost of treating pneumonia approximated that of the national hospital and differed from the remaining district hospitals.

In line with earlier studies meningitis was the most expensive condition to treat [22,23]. However, the recently estimated cost of US$ 2,043 per case of meningitis treated in Pakistan is six-fold higher than the estimates we have reported [22]. This is likely to reflect the difference in health care setting and the range of investigations and drugs used in the treatment of meningitis that significantly impact total costs. In our sample, treatment was predominantly based on inexpensive first line drugs and in most sites laboratories conducted only basic aspects of CSF analysis, and very rarely any imaging investigations. For malaria and pneumonia our estimates are similar to those appearing in the literature. When adjusting the 1993/4 study of malaria treatment costs in 2 Kenyan district hospitals [13] to 2005 values, the average costs range from US$ 46 to US$ 63, depending on disease severity, which is very comparable to our provider cost estimates of between US$ 47 and US$ 75 for treatment of malaria in a district hospital. The range of treatment costs we report (US$ 46–U$ 172) for pneumonia compares with the average cost of US$ 71 per episode of pneumonia and US$ 236 for severe pneumonia reported recently in Pakistan [22].

An important finding was that rationalising resource use can have a significant impact on the cost of treatment. Firstly, national treatment guidelines for severe pneumonia recommend that inpatients be treated with a single antibiotic only and those with very severe disease receive a combination of two antibiotics. However, over half the children with pneumonia as a stand-alone diagnosis received at least 4 drugs. Adherence to clinical guidelines and rational resource use could be effective in reducing costs of treatment. We found that the mean cost of a drug prescription for a child with pneumonia across the hospitals was between US$ 3.0 – US$ 31.2, but the mean cost for essential drugs only in this sample would be US$ 0.36 for severe pneumonia and US$ 0.9 in a case of very severe pneumonia. Secondly, the inability of households to raise user charges in time resulted in prolonged hospital stay and additional costs, especially at tertiary referral. The opportunity costs of having children that have already been discharged occupying limited hospital beds is considerable, so there is an urgent need for change in practice in this area.

The finding of high user charge is not surprising for two reasons. Patients admitted within health facilities commonly fail to raise the charges requested on discharge in both public and private facilities. Secondly, a report of user fees charge in Kenyan faith-based health institution has reported 120% cost recovery for treatment costs of childhood malaria [24]. Such high recoveries cover cost deficits for more expensive to treat conditions.

Similar to an earlier report by the ministry of health [9], household earnings and personal savings were the most common sources of payment in our study. The National Hospital Insurance Fund (NHIF) set up in 1996 provides medical insurance to employed Kenyans earning more than US $13.29 (KES 1000) per month [25]. The fund currently has a membership of 1.8 million out of 14.9 million people in the 15 to 65 years age group [25], implying most Kenyan household are not covered. These households pay for their healthcare directly from their earnings and savings.

The findings reported have a number of limitations. The estimates we used for bed-day cost of hospitals are based on results of two studies each representing a single hospital in Kenya and it is known that bed-day costs vary significantly between hospitals within countries, especially due to differences in occupancy rates [15]. Unfortunately, overcoming this limitation is difficult given the expense of conducting multiple formal hospital costing studies. Secondly, pre-admission costs were underestimated in our study as outpatient facilities were not included. Future studies should extend such analyses to cover children attending outpatient facilities, as this will provide more comprehensive information on the cost of treatment.

## Conclusion

The treatments cost estimates we report vary by facility type and significantly increase the body of data available in Kenya. These costs can be interpreted as approximate values for disease specific treatment costs in similar facility types in Kenya, and perhaps similarly placed low-income countries. It was revealed that the tertiary public facility charged similar levels of user fees as a mission hospital included in the analysis and that these payments were a major challenge to households. The second mission hospital recovered entire provider costs by charging user fees.

## Competing interests

The authors declare that they have no competing interests. Those funding the work had no role in study design, reporting or the decision to submit the manuscript for publication.

## Authors' contributions

PA participated in the analysis and wrote the manuscript. AOA was responsible for data collection and participated in cost analysis. UKG designed the study, participated in the cost analysis and in writing the manuscript. ME conceived of the study, oversaw its design and coordination, and participated in writing the manuscript. All authors read and approved the final manuscript.

## Supplementary Material

Additional file 1 **Study questionnaire**. Caregiver and Out-of-Pocket Costs Questionnaire. Click here for file
