# Supplementary material for: The economic burden of inpatient paediatric care in Kenya: household and provider costs for treatment of pneumonia, malaria and meningitis
Source: Cost Eff Resour Alloc. 2009 Jan 22;7:3. doi: 10.1186/1478-7547-7-3 (PMC2640355; doi:10.1186/1478-7547-7-3)
Supplement: Additional file 1 — Study questionnaire. Caregiver and Out-of-Pocket Costs Questionnaire. [file 1478-7547-7-3-S1.doc]

**Study questionnaire**

### Caregiver and Out-of-Pocket Costs Questionnaire

*Guidance to interviewer: For questions with more than one possible answer, tick the relevant box* ***and*** write the code number on the line below the question.

|  | **Facility information** | | |
| --- | --- | --- | --- |
| 1. | Facility name: | _________________________ | |
| 2. | Type of facility: | - National Hospital - Provincial Hospital - District Hospital - Mission Hospital | |
| 3. | Facility study ID code: | |  |  | | --- | --- | | |
|  |  |  | |
|  | **Patient information:** |  | |
| 4. | Patient study ID | ­­­­­­­­­  __________________________ | |
| 5. | Patient ID from patient records: | ___________________________  Gender:   - Male - Female | |
| 6. | Date of birth: | |__|__| |__|__| |__|__|__|__|  day month year  (age in yrs / mths / days if dob not available) | |
| 7. | Informed Consent Date: | |__|__| |__|__| |__|__|__|__|  day month year | |
|  | **Caregiver Information:** |  | |
| 8. | Relationship to the patient:  _______________ | - Mother = 1 - Father = 2 - Sister = 3 - Brother = 4 - Grandmother = 5 | - Grandfather = 6 - Other relative = 7 = 7 - Friend = 8 - Other (specify) = 9 |

|  | **Travel Information** | |  | |
| --- | --- | --- | --- | --- |
| 9. | How long did it take to get here from your home (including the journey time and any waiting for transport)? | | Minutes |___|___|  Hours |___|___|  Unknown | |
| 10. | What kind of transportation did you use to bring your child to this hospital or clinic?  *In case of multiple means of transportation during this trip, please tick only the transportation that was used for the longest distance).* | | - Car = 1 - Bus / train = 2 - Bicycle = 3 - Motorbike = 4 - Taxi = 5 - Ambulance = 6 - Boat = 7 - By foot = 8 - Other, specify: = 9   ___________________ | |
| 11. | If you paid for transportation to bring the child to the hospital or clinic, how much did you pay?  Ksh_________________ *(put* ***0*** *if no payment was made and 999 if don't know)* | |  | |
| 12. | How many trips did you or other household members make to visit your child?  *(Total numbers of round trips)*  *Examples: 3 relatives' visit one time [****n = 3 trips****] One relative visits three times [****n = 3 trips****]* | | |___|___|  (Put ***0*** *if no visit was made)* | |
| 13. | What kind of transportation did ***you*** use to come to this hospital or clinic to visit your child?  *(It concerns the last used transportation that has been used to visit your child).*  *______________________* | | - Car = 1 - Bus / train = 2 - Bicycle = 3 - Motorbike = 4 - Taxi = 5 - Boat = 7 - By foot = 8 - Other, specify = 9 | |
| 14. | If you paid for transportation, how much did ***you*** pay to visit this health care facility?  *(Round trip, one person) If you used different means of transportation, please choose the most oftenly used.* | | Ksh _________________  *(put* ***0*** *if no payment was made and 999 if don't know)* | |
|  | | **Treatment Costs** | |  |
| 15. | | Before visiting this facility, did you seek help from any of the following? How much did it cost you for drugs, tests, consultation and other financial costs? (**Caretaker to list all the facilities visited, then ask the cost of each item for each place visited one at a time**)   | Expenditure/ Facility | Hospital | Priv clin | Pub clin | Pharmacy | Traditional healer | Friend | Shop | Other | | --- | --- | --- | --- | --- | --- | --- | --- | --- | | Drug |  |  |  |  |  |  |  |  | | Diagnostic test |  |  |  |  |  |  |  |  | | Consultation |  |  |  |  |  |  |  |  | | Other financial cost |  |  |  |  |  |  |  |  | | Total cost |  |  |  |  |  |  |  |  |   Key Priv clin = Private clinic, pub clin = Public clinic | | |
| 16. | | How much did the household pay for drugs,tests, consultation, and other fees for this visit or hospitalisation?   | Item | Drug | Tests | Consultation fee | Other fees | Bed charges | Total | | --- | --- | --- | --- | --- | --- | --- | | Cost  *(put* ***0*** *if no payment was made and 999 if don't know)* |  |  |  |  |  |  | | How much were you required to pay? |  |  |  |  |  |  | | | |
| 17. | | Are you losing some income from being here?  ⬜ Yes  ⬜ No | | |
| 17b.. | | If you weren’t here today, what would you be doing?  ⬜Nothing = 1  ⬜ Housework = 2  ⬜ Looking after my children = 3  ⬜ Working (specify)______ = 4  ⬜ Other (specify)________ = 5  ⬜ Don’t know = 6 | | |

| 18. | How much income have you or other family members lost as a result of taking care of your child instead of working?   | PERSON | YOU | FAMILY  MEMBER 1 | FAMILY  MEMBER 2 | FAMILY  MEMBER 3 | | --- | --- | --- | --- | --- | | AMOUNT  LOST (KSH) |  |  |  |  | | |
| --- | --- | --- | --- | --- | --- | --- | --- | --- | --- | --- | --- | --- |
|  |  | |
|  | **Financing of the costs of treatment and transport** | |
| 19. | Has the illness affected the family financially? | - Yes - No |
| 20. | Where did the money come from to pay for these expenses?  (Multiple responses allowed) | - Cutting down on other expenses = 1 - Using savings = 2 - Borrowing = 3 - Selling assets = 4 - Asking for donations from friends and relatives = 5   Others, specify = 6 |
| 21. | What is the total number of people in your household? | _____ Adults ______ Children |
| 22. | What are the total expenses of the household where the child lives, including rent and other "fixed" expenses?   | Item/ amount | Per day | Per week | Per month | | --- | --- | --- | --- | | Food |  |  |  | | Education |  |  |  | | Rent |  |  |  | | Household item |  |  |  | | Total |  |  |  | | |

| 23. | This form was completed by: ______________________________  Date |__|__| |__|__|__| |__|__|__|__|  day month year  **Date of admission**  |___|___| Date |___|___| Month |___|___| Year  **Date of discharge / referral**  |___|___| Date |___|___| Month |___|___| Year |
| --- | --- |
